# Use of Average Mutual Information and Derived Measures to Find Coding Regions

**DOI:** 10.3390/e23101324

**Published:** 2021-10-11

**Authors:** Garin Newcomb, Khalid Sayood

**Affiliations:** Department of Electrical and Computer Engineering, University of Nebraska, Lincoln, NE 68588-0511, USA; garin.newcomb@huskers.unl.edu

**Keywords:** mutual information, DNA annotation, protein coding

## Abstract

One of the important steps in the annotation of genomes is the identification of regions in the genome which code for proteins. One of the tools used by most annotation approaches is the use of signals extracted from genomic regions that can be used to identify whether the region is a protein coding region. Motivated by the fact that these regions are information bearing structures we propose signals based on measures motivated by the average mutual information for use in this task. We show that these signals can be used to identify coding and noncoding sequences with high accuracy. We also show that these signals are robust across species, phyla, and kingdom and can, therefore, be used in species agnostic genome annotation algorithms for identifying protein coding regions. These in turn could be used for gene identification.

## 1. Background

The success of sequencing technologies and the resulting explosion of available sequence information has made the task of annotating genomic sequences both more important and more challenging. One of the major annotation tasks is the identification of protein coding genes. This task has become especially important because of the increasing risk of the spread of infectious diseases and the increasing opportunities afforded to molecular medicine. Identifying genes in pathogens can tell us much about their potential impact and the possible avenues of control; identifying genes and their interactions can lead to understanding of disease as well as possible therapeutic pathways.

Computational gene finding techniques are divided into similarity based approaches, ab initio approaches, and hybrid approaches. Similarity based approaches rely on the existence of similar sequences in a database; the annotation of the sequence in question can be guided by the annotation of any similar sequences in the database. Aligning the sequence to be annotated with an already annotated sequence can help identify functional regions in the sequence to be annotated [1,2,3]. As the number of the sequences in the database grows such comparative methods provide a quick and efficient way of identifying functional elements such as protein coding regions. Ab initio methods rely on patterns in the genomic sequence which signal the presence of a gene. These can be patterns of nucleotides or amino acids identified from existing genomic and amino acid databases [4], it could be structural signals such as known promotor regions or CpG islands [5], it could be statistical patterns dependent partially on the triplet nature of coding regions. The statistical patterns are often captured using Markov models [6,7,8].

As the structure of genes is different for prokaryotes and eukaryotes the ab initio methods for gene finding in prokaryotes and eukaryotes generally differ as well. A sequence beginning with a start codon and ending with a stop codon is called an open reading frame or ORF. If the nucleotide sequence was random a stop codon will show up about every fifty nucleotides so if we have a much longer sequence of nucleotides which begin with a start codon and ends with a stop codon it is likely to be a coding sequence. In prokaryotes where the gene is usually a continuous sequence of nucleotides the ORF is often also the gene. In eukaryotes stop codons may be present in introns as well so for a multi-exon gene we would look for sequences bound by a start codon and a boundary between an exon and an intron (i.e., a splice site), regions bound by splice sites, or a region bound by a splice site and a stop site. Gene finding involves a number of operations. Putative regions that might be protein coding genes may be flagged by identifying the location of start, stop, and splice sites, by looking for promoter regions [9], by identifying CpG islands which tend to occur upstream of genes [5]. Once a putative coding region is identified the region and their surroundings can then be tested for other signals that may indicate that it is a coding region. These signals include codon composition as there are codons that occur with higher frequency in some species, hexamer composition [10], nucleotide composition, and periodic occurrence of bases. Another popular method for detecting coding regions is through the use of Markov models. Hidden Markov Models are used in a number of gene prediction software including the popular GenScan [10]. Interpolated Markov models form the basis for the GLIMMER tools [11,12,13].

Coding regions in genes are information bearing structures which is why Markov models have been the workhorse for identifying coding regions. It thus makes sense to see if measures of information can be used as signals for distinguishing coding and noncoding regions. One measure of the information structure of genomes is the average mutual information (AMI) profile [14,15,16] which is based on the information contained in a base about another base *k* bases away. The average mutual information profile looks at the linear and nonlinear dependencies between residues separated by different lags. The average mutual information profile has been used to generate phylogenetic trees at all levels of the tree of life. The effect of the differences between coding and noncoding regions is visually apparent when we look at AMI profiles of prokaryotes, whose genomes primarily consist of coding regions, and animals where only a small portion of the genome consists of coding regions. However, attempts at using the AMI profile to distinguish between coding and noncoding regions are not particularly successful as we shall see in the results section. However, we show that if we remove the averaging effect of the average mutual information profile by constituting its components to form a new profile we are substantially more successful in using the resultant vector as a signal for discriminating between protein coding regions and non protein coding regions.

In this work we show that the signals generated by these derived metrics are sufficiently robust that they can be used in a universal manner to identify coding regions in organisms throughout the tree of life. While the simplicity of the signals allows for easy incorporation in high throughput gene prediction pipelines, we are not presenting such a pipeline. Our more modest goal is to provide a computational signal that provides effective discrimination between protein coding and noncoding regions in a robust manner. This robustness allows these signals to be used to explore genomic fragments of unknown origin. A major contribution of this work is that unlike most known computational signals used in gene prediction pipelines the signals generated by the proposed metrics are not only highly discriminatory with most The area under the curve (AUC) values greater than 0.9, they also provide robust discrimination when they are applied to species other than the ones used to obtain the signals. This is true even when the other species belong to different kingdoms. The availability of robust signals which can provide cross-species prediction can help remove a significant barrier for exploration of novel genomes.

The MATLAB programs used to generate the results presented in this work are available at the GitHub repository, AMICodingRegionPrediction, https://github.com/gnewcombUNL/AMICodingRegionPrediction (accessed on 7 August 2021). We have also included a detailed example on how to use the program and modify parameters to accommodate the interests of the users.

## 2. Average Mutual Information and Its Derivatives

### 2.1. Average Mutual Information

Genomic sequences are information bearing sequences and hence the arrangement of nucleotides in the sequence is not random. This deviation from randomness reflects biological constraints. There are a number of ways to measure this structure in genomes including correlation analysis [17], complexity measures [18,19], and various information theoretic measures [20]. One information theoretic measure that has been particularly effective for identifying structural similarities between genomes of evolutionarily related organisms has been the average mutual information.

The information associated with an event depends on the uncertainty associated with the event. The average uncertainty is quantified by the Shannon entropy [21], H(X), which is defined as:

(1) $$H\left(X\right)=-\sum_{x\in A}p\left(x\right)\log p\left(x\right)$$

where A is the alphabet which makes up the sample space for the discrete random variable *X*. In our current application the alphabet consists of the letters denoting the nucleotides adenine, cytosine, guanine, and thymine.

A={A,G,C,T}

This is easily extended to multiple events. Events which influence each other have mutual information. This means that knowing the outcome of one event provides information about the other. That is, the uncertainty concerning the latter event is reduced. Average mutual information (AMI), I(X;Y), measures the information contained in event *X* about event *Y*, and is defined as:

(2) $$\begin{array}{cc} I(X;Y) & =H(X)-H(X|Y)\\ \; & =\sum_{X\in A}\sum_{Y\in A}p\left(X,Y\right)\log\frac{p(X,Y)}{p(X)p(Y)} \end{array}$$

While the principal use of average mutual information is in the area of communication and data compression [22] it has been used in a variety of fields, including bioinformatics. Specifically, it has been used to study the covariation of residues in the envelope protein of HIV [23] and other proteins [20,24]. It has also been used to aid in sequence assembly [25], and to generate a species-specific signature [16] for phylogenetic analysis. Previously, whole and partial genome AMI profiles have been used to classify fungal and mycobacterial samples [26], study changes in HIV populations [27], and study genomic signatures in viral sequences [28]. While there have been indications that the average mutual information could be used to differentiate between coding and noncoding sequences [14,29] the attempts to do so have not been particularly successful. In this work we show that while the average mutual information profile as it has been previously used is not a very strong signal for coding regions we can derive metrics from it that are stronger signals for protein coding regions than even the highly popular interpolated Markov models.

Let the discrete random variable *X* correspond to a nucleotide at an arbitrary location *n* and the discrete random variable *Y* correspond to a nucleotide at location n+k. We refer to *k* as the lag. The random variables *X* and *Y* take on values from the alphabet: A={A,C,G,T}. We can estimate the marginal probability distributions p(X) and p(Y) by counting the number of times each nucleotide occurs divided by the length of the sequence. In other words the probability that *X* takes on the value *A* is estimated by counting the number of times *A* occurs in the sequence divided by the length of the sequence. The other probabilities are estimated in the same manner. Note that because we are not taking into account the locations of the nucleotides the estimates for p(X) and p(Y) are the same, since both are measured across the entire sequence. We call this estimate ${\hat{p}}_{0}\left(X\right)$. Similarly, the joint probability distribution p(X,Y) is estimated by counting the number of times each of the 16 possible pairs of nucleotides separated by *k* base pairs occurs, and dividing by the total number of such pairs in the sequence. We call this estimate ${\hat{p}}_{k}\left(X,Y\right)$ for lag *k*. Using these probability estimates, we generate an AMI profile $AMI_{k}$ for selected values of *k* as follows:

(3) $$AMI_{K}=\sum_{X\in A}\sum_{Y\in A}{\hat{p}}_{k}\left(X,Y\right)\log\frac{{\hat{p}}_{k}\left(X,Y\right)}{{\hat{p}}_{0}\left(X\right){\hat{p}}_{0}\left(Y\right)}$$

If the nucleotide occurring at position n+k is independent of the nucleotide at position *n*, then the average mutual information between the two events is 0 (i.e., $AMI_{K}=0$). Likewise, if there is a peak in the AMI profile at some lag *k*, this indicates increased dependency between nucleotides *k* base pairs apart.

### 2.2. Average Mutual Information Derivatives

The AMI profile provides a glimpse into how a sequence’s base pairs bias surrounding base pairs at particular lags. A less succinct profile that provides additional information can be defined by collecting the individual terms that are summed when calculating AMI. We call this profile “expanded adjusted Average Mutual Information” (eaAMI). For each value of *k*, the profile consists of 16 elements, one for each possible pair of nucleotides *k* bases apart. The frequency of each nucleotide pair is estimated, and then scaled by the dependence between the two nucleotides. That is, the profile element for lag *k* and nucleotide pair X,Y is defined as:

(4) $$eaAMI_{K}\left(X,Y\right)={\hat{p}}_{k}\left(X,Y\right)\log\frac{{\hat{p}}_{k}\left(X,Y\right)}{{\hat{p}}_{0}\left(X\right){\hat{p}}_{0}\left(Y\right)}$$

Thus, the profile consists of 16k values, concatenated into a single vector.

A slightly simpler profile utilizes the unadjusted nucleotide pair frequencies, and is thus termed the “expanded Average Mutual Information” (eAMI). Formally, each profile element is defined as:

(5) $$eAMI_{K}\left(X,Y\right)={\hat{p}}_{k}\left(X,Y\right)$$

As with eaAMI, the eAMI profile consists of 16k values. These AMI variants are easily extended to use with amino acid sequences, which may provide different (but overlapping) information about the sequence.

## 3. Prediction Methodology

The numerical profiles defined above provide a mechanism to map a nucleic acid sequence into a vector space that is readily analyzed and manipulated. Each profile presented here defines a different space, but the techniques used to operate on the space are generic. We use linear Support Vector Machines (SVMs) to perform binary classification. As with any classification problem, data is partitioned into training sets and test sets. In order to objectively measure the methods’ performance, the data sets are intentionally contrived. The data sets are generated from a repository of 82 species with well-annotated genomes. The full list of species is included in the Supplementary Materials. Each species is identified by its taxonomic ID. We downloaded the genomic FASTA and GTF files for that assembly from NCBI. For each GTF file, we compiled all annotated protein coding regions, denoted “CDS” (Coding DNA Sequence) in the annotation. We accepted the annotation as-is and did not speculate on the possible existence of unannotated CDSs. For large eukaryotic genomes, we used only the first three chromosomes. Given the CDS coordinates, we extracted each from the corresponding genome, taking the reverse complement of each CDS on the negative strand. All CDSs were then concatenated into a parent coding sequence, such that there was a single coding sequence for each species. These range from 500 thousand base pairs to tens of millions of base pairs. We then extracted the noncoding sequences. Any segment of the genome that is not included in any CDS on either strand was considered to be noncoding. Noncoding regions from both strands were included so that noncoding regions surrounding CDSs on both strands are represented. These were all extracted and concatenated into a single parent noncoding sequence for each species. These range in length from 60 thousand base pairs to hundreds of millions of base pairs

The data sets are then constructed by randomly drawing 2000 non-overlapping sequences of constant length from each of the two parent sequences. Each data set used to train and test an SVM includes sequences of constant length, but multiple data sets were constructed with a different length for each. If the genome is too short to allow for 2000 non-overlapping sequences at the given length, the number of sequences in the data set is reduced accordingly. This allows us to evaluate the effect of sequence length on predictive performance for a range of 25 to 10,000 base pairs.

We use k-fold cross-validation, with k = 5. The SVM is trained on the training folds, and its performance evaluated by using it to classify the test folds. The output of the SVM classifier is a score assigned to each input profile. The magnitude of the classification score is the distance from the profile to the SVM decision boundary, and the sign specifies on which side of the boundary the profile falls. Positive scores indicate profiles on the side of the boundary corresponding to the coding region class. A higher score indicates a higher probability that the test sequence was drawn from the coding region parent sequence. We also evaluate a Euclidean distance classifier. Given a training set of sequence profiles from coding and noncoding regions, we calculate the centroid for both sets. For each sequence in the test set, we determine the Euclidean distance to both centroids, and subtract one from the other to determine classification scores. Positive values indicate that the test sequence profile is closer to the coding region centroid. Scores produced by each classifier for each type of profile are used to evaluate the classification performance.

In practice, when predicting coding regions for an unannotated genome, we would need to use a model trained on some other organism. We evaluate this cross-species scenario by training a model for each organism we consider, and using the model to predict coding regions in all other organisms.

## 4. Results

Receiver Operating Characteristic (ROC) curves are generated by sweeping a prediction threshold across the entire range of scores for each prediction methodology. That is, each score that is produced by the SVM is used as a threshold to generate a point on the ROC curve. For each threshold, sequences that score higher than the threshold are declared to be coding regions, while those that score lower are declared to be noncoding regions. We then calculate the true and false positive rates, which yields a point on the ROC curve. This is repeated for all scores produced by the SVM.

**Figure 1:**
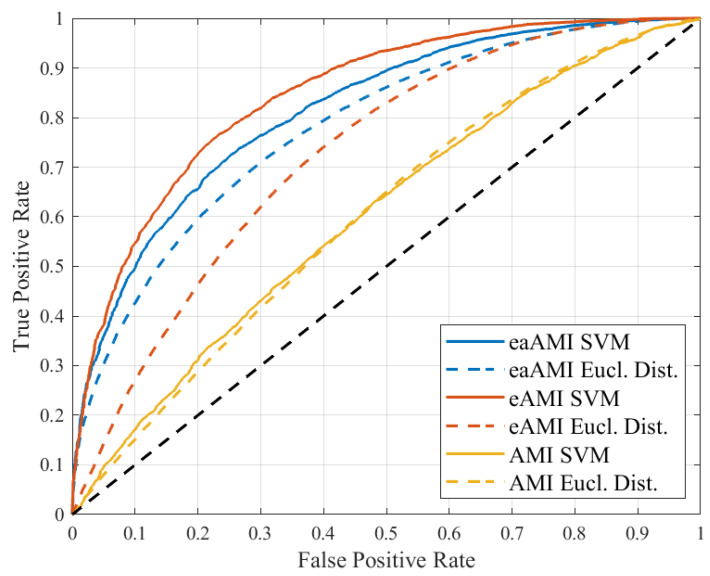
ROC curves for coding region prediction using SVMs on AMI profiles with length 100 bp sequences drawn from the *S. cerevisiae* genome.

Sample curves for the organism *S. cerevisiae* are shown in Figure 1. The area under the curve (AUC) is then used as an objective single-value metric for evaluating prediction performance. Additionally, the classifier’s sensitivity and specificity are calculated using a threshold of 0. That is, coding regions assigned a positive score by the SVM are considered true positives, while noncoding regions assigned a negative score are considered true negatives. All quoted values of sensitivity and specificity that appear in this paper use this 0 threshold.

Increasing the information available to the classifiers yields AUC improvements. As is to be expected, the improvement tapers as *k* continues to increase. For eAMI, there are diminishing returns for *k* values greater than 2. The ROC curves are reasonably symmetric about the line y=1−x. This suggests that at the optimal decision threshold (the point on the ROC curve closest to a 0% false positive rate and 100% true positive rate), the false positive and true positive rates will be balanced.

**Figure 2:**
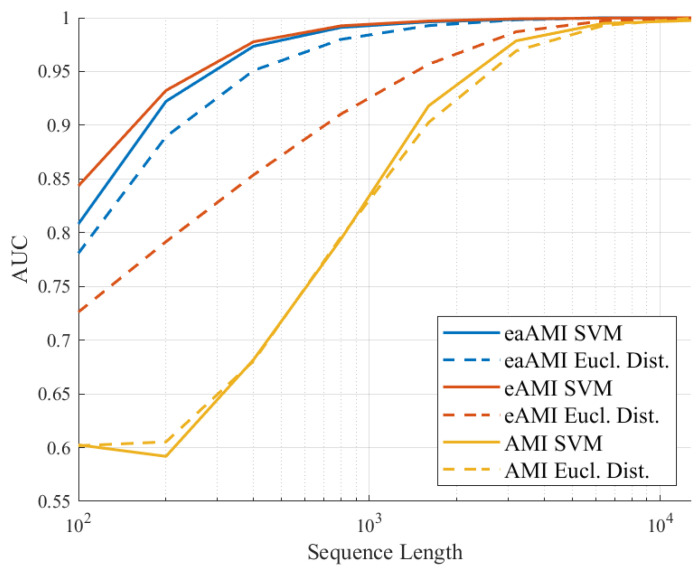
AUC for coding region prediction using SVMs and Euclidean distance on AMI profiles derived from sequences of increasing length drawn from the *S. cerevisiae* genome.

The longer a sequence is, the more closely it will tend to resemble the aggregate profile of the set to which it belongs. Accordingly, we would expect prediction performance to increase as sequence length increases. To evaluate this effect, we constructed data sets consisting of sequences of increasing length. This is shown for *S. cerevisiae* in Figure 2 for the proposed profiles. We can see that while the results for each profile is different, in each case the SVM classifier works better than the simple Euclidean distance classifier. For AMI, it does no better than Euclidean distance, but there is a significant performance premium for eAMI, and a small but meaningful gain for eaAMI. For the remainder of this work we only present results using the SVM classifier.

### 4.1. Profile Analysis

For all profiles considered here, we can effectively distinguish between coding regions and noncoding regions provided we have a sequence of sufficient length. This suggests that profiles for members of both classes converge to some characteristic profile. To best represent these characteristic profiles, we calculate the centroid of profiles drawn from the longest sequences considered in this analysis (12,800 base pairs). These centroids provide a visual representation of the differences between coding and noncoding regions, from which we can glean features about the class from which they were drawn.

**Figure 3:**
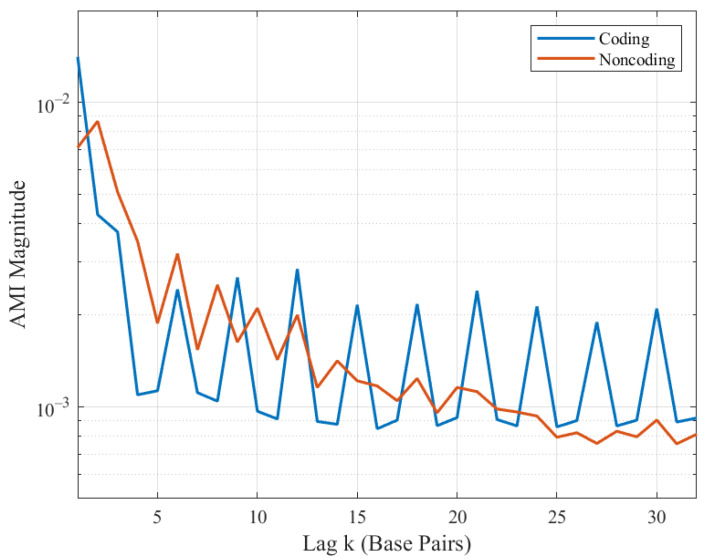
Centroid AMI profiles for *S. cerevisiae* coding and noncoding regions.

Centroid AMI profiles are presented in Figure 3. The most obvious feature is the presence of distinct peaks at multiples of three in coding regions, resulting from the triplet periodicity conferred by codon abundance biases. The notable exception to this periodicity is the inflated values for lags 1–2, suggesting biases in the occurrences of certain nucleotide pairs and triplets. This is possibly due to the fact that most amino acids are encoded by a number of different triplets or synonymous codons [30]. Different organisms show a bias to one of the synonymous codons resulting in their higher abundance in the coding sequence. For lags greater than 4, magnitude decreases only slightly over the window considered here. The noncoding centroid also has noticeable, if less pronounced features. It too is marked by significant magnitudes for small lags, but with more gradual degradation thereafter. Curiously, there appear to be small but significant peaks at even lags from 6 to 14.

**Figure 4:**
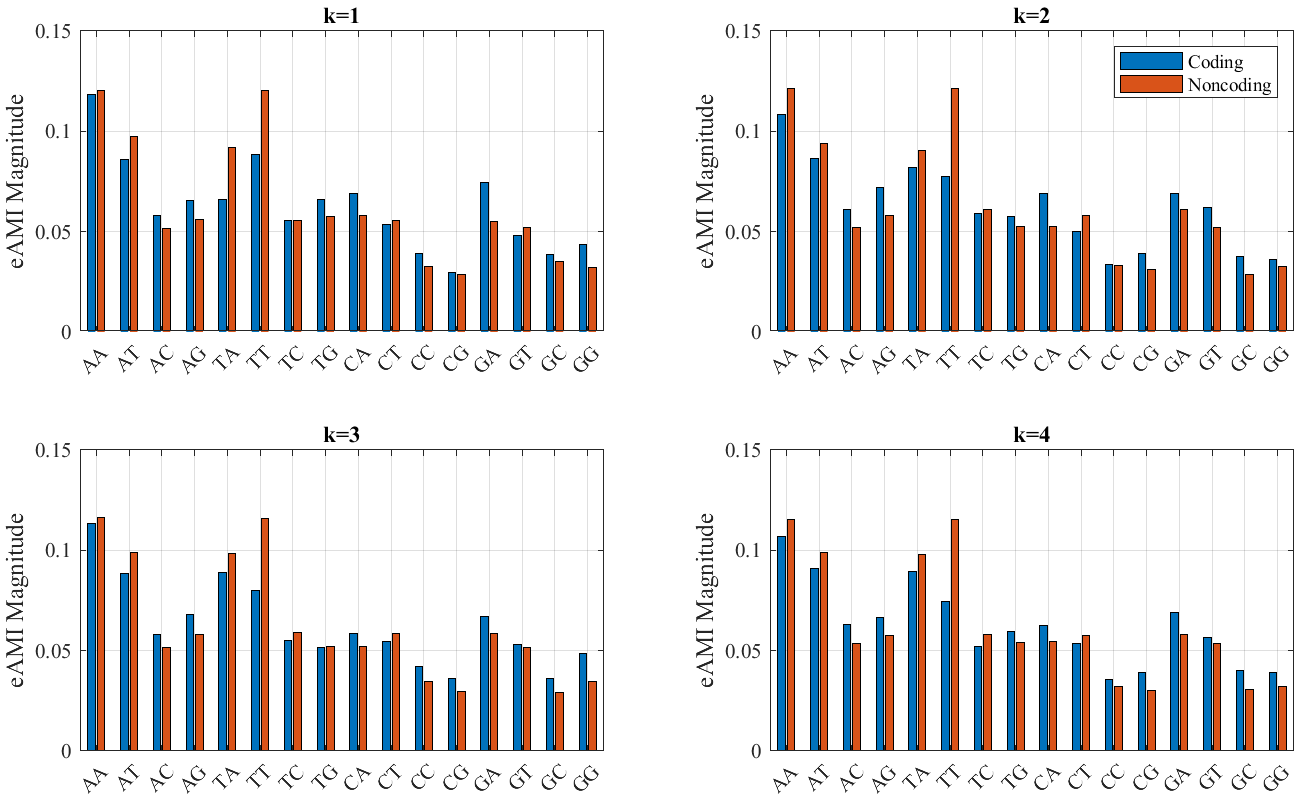
Centroid eAMI profiles for *S. cerevisiae* coding and noncoding regions.

Centroid eAMI profiles are presented in Figure 4 for lags 1–4. Subsequent lags are omitted for brevity, but they bear resemblance to those presented. The presence of strings of thymine is most indicative of a noncoding region. This would occur in the opposite strand of a poly(A) tail downstream of a coding region. Noncoding regions are more symmetric, in the sense that complementary nucleotide pairs have similar abundance. This is due to the relatively higher likelihood of the opposing strand of a noncoding region also being noncoding.

**Figure 5:**
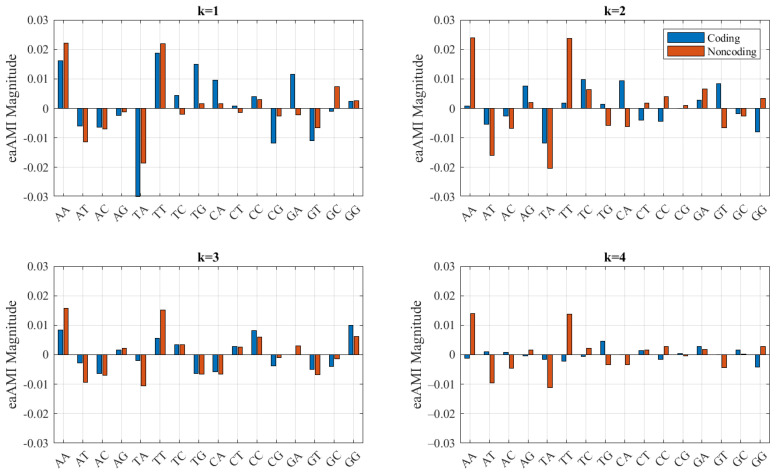
Centroid eaAMI profiles for *S. cerevisiae* coding and noncoding regions.

Centroid eaAMI profiles are presented in Figure 5 for lags 1–4. Again, subsequent lags are omitted for brevity.

### 4.2. All Species Predictions

**Figure 6:**
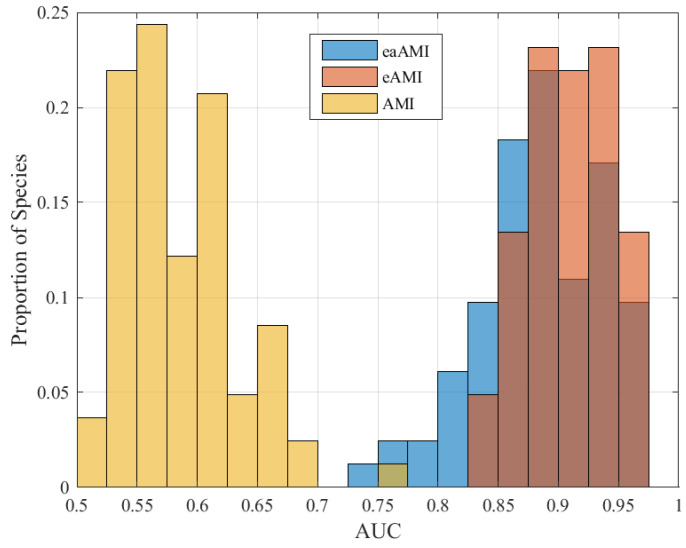
AUC distribution for all three profiles across all 82 species.

In order to evaluate the robustness of this method, we applied it to 82 genomes, consisting of 70 bacteria, 1 archaea, and 11 eukaryotes. For length 100 base pair sequences, eAMI produced the highest AUC in 78 of the 82 species considered. AUC results for all profile types and species are summarized in the histograms in Figure 6. The eAMI SVM performed best for the lone archaea, *M. maripaludis* (0.971 AUC, 92.7% sensitivity, and 89.6% specificity), and worst for *A. nidulans* (0.826 AUC, 80.5% sensitivity, and 68.7% specificity).

**Figure 7:**
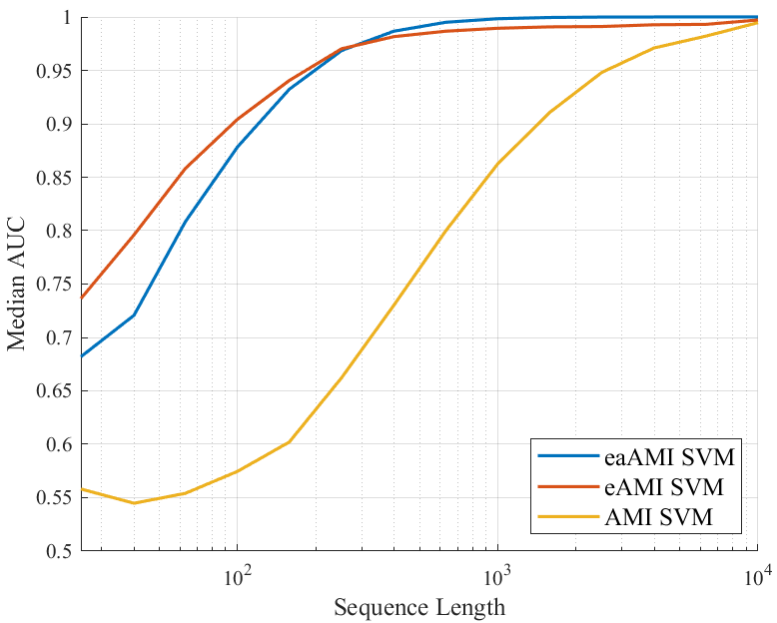
Median AUC values for all three profiles as a function of the length of the sequence being classified.

**Figure 8:**
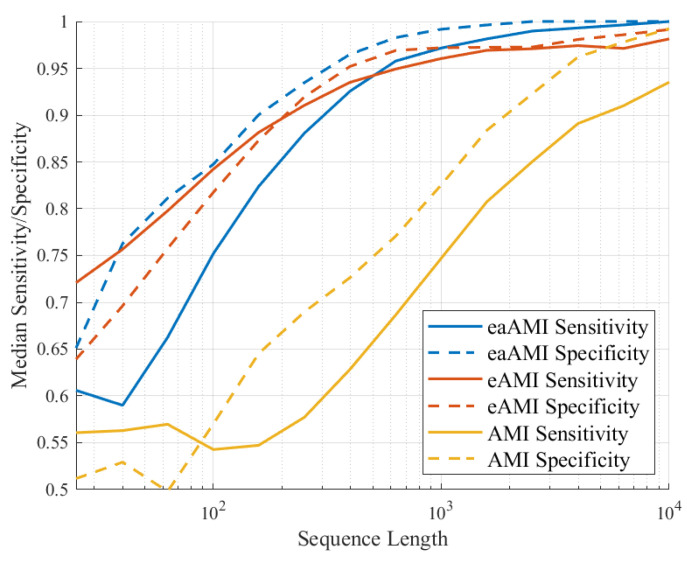
Median sensitivity and specificity values for all three profiles as a function of the length of the sequence being classified.

As would be expected from the results in Figure 2 for *S. cerevisiae* increasing the length of the sequences results in better performance as can be seen in Figure 7 and Figure 8.

We can see that while the AUC, sensitivity, and specificity values for classifiers using the AMI metric perform poorly for much of range of lengths, the performance of the classifiers using the eaAMI and eAMI metrics perform well for a wide range of lengths, the performance improving with the length of the sequence. Comparing the performance using eaAMI and eAMI profiles we see that the latter performs better for sequences of length less than 250 bp wile the former performs better for all lengths greater than 250 bp.

### 4.3. Cross-Species Predictions

To see the robustness of this approach to training based on organisms from different branches of the tree of life—other species, phyla, and kingdoms—we show the AUC, sensitivity, and specificity of the classification when using training sets from widely different organisms. The complete results are available in the Supplementary Materials. We compare these results to results obtained using the widely used Glimmer package under identical conditions. In order to get these results we used “build-icm” to train a model, and ”glimmer3” to score the test set. We used the “-separate_genes” option, which interprets the input file as a set of (potential) genes to score as such, rather than a genome fragment to be scanned for ORFs that are then scored. We use the “Raw Score” to compute AUC, sensitivity, and specificity.

**Figure 9:**
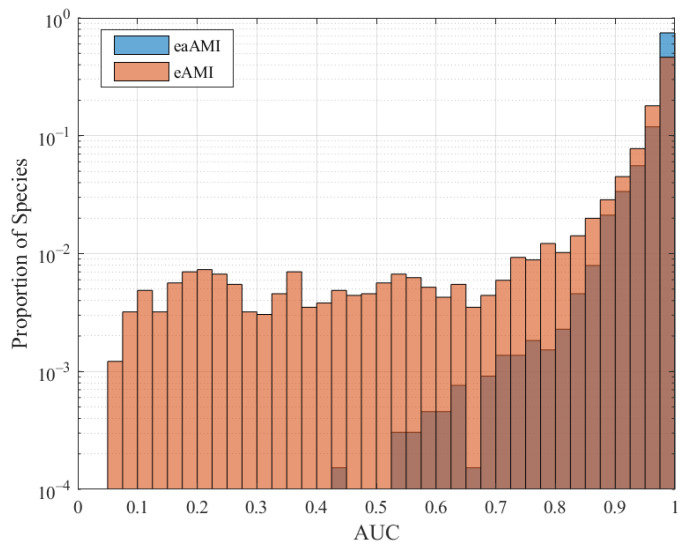
AUC distribution for eAMI and eaAMI profiles across all pairwise cross-species predictions.

AUC results for all proposed profile types and species are summarized in the histograms in Figure 9 for length 1000 base pair sequences. Interestingly, eaAMI produces better results on average, and is also considerably more consistent. A total of 95.4% of cross-species predictions made using eaAMI profiles resulted in AUC greater than 0.9, compared with 76.6% of predictions using eAMI profiles.

**Figure 10:**
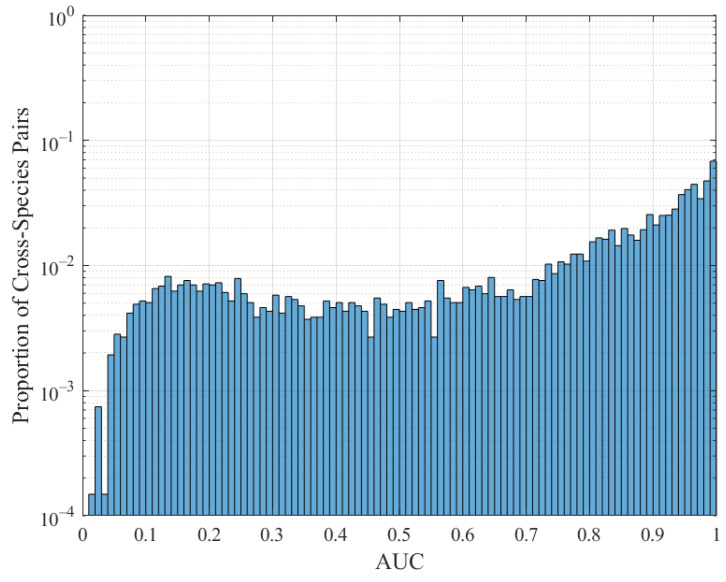
AUC distribution for Glimmer across all pairwise cross-species predictions.

The corresponding AUC results for Glimmer are shown in Figure 10.

**Figure 11:**
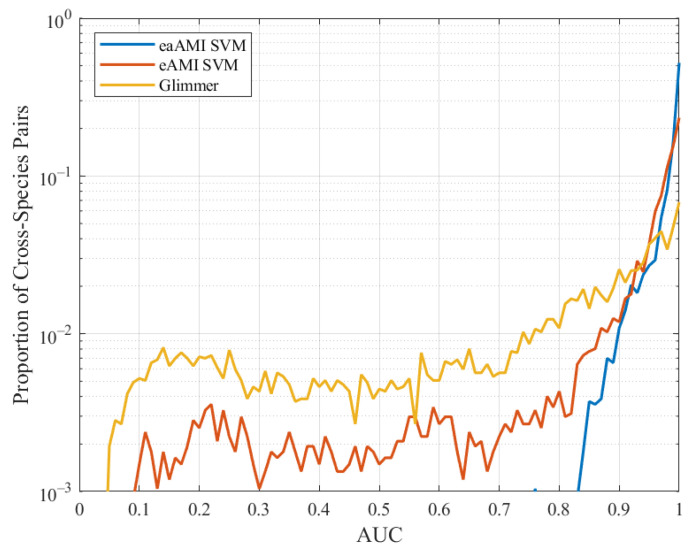
AUC distribution for Glimmer, eAMI, and eaAMI across all pairwise cross-species predictions.

The classification performance using the latest version of Glimmer is better than the classification performance using AMI, slightly worse than the classification performance using eAMI, and considerably worse than the classification performance using eaAMI. We compare the classification performance of Glimmer with the performance using eAMI and eaAMI in Figure 11.

Median AUC, sensitivity, and specificity for each profile (including Glimmer) for two different sequence lengths are shown in Table 1.

Glimmer outperforms the classifier using the AMI profile and is considerably outperformed by the eaAMI and eAMI profiles regardless of sequence length.

Given the superior performance of the eaAMI signal we sample some of the results using eaAMI to show the robustness of this particular signal to differences in the species, phylum, or kingdom of the organism used for training and the one used for testing. The complete results for all cross-species predictions are available in the Supplementary Materials.

In Table 2, we show the results when using different organisms in the fungal genus *Aspergillus* for training and testing. The first number in the table is the AUC, the second is the sensitivity, and the third is the specificity.

We compare these results with those obtained using the latest version of Glimmer in Table 3.

Notice that while the performance metrics for Glimmer are close to the performance metrics using eaAMI when we use the same species for training and prediction they are worse when we use different species for training and prediction.

To see how the performance changes when we use organisms belonging to different orders we use four Gammaproteobacteria: *Acinetobacter baumanii* belonging to the Pseudomondales order, *Streptococcus pneumoniae* belonging to the Lactobacillales order, *Salmonella enterica* belonging to the Enterobacter order, and *Vibrio cholerae* belonging to the Vibrioles order. The results are shown in Table 4.

As we can see there is remarkable consistency in classification performance.

However when we use Glimmer under the same conditions there is a significant drop in performance as we can see from Table 5.

Finally, let us look at what happens if we use DNA sequences from organisms belonging to different kingdoms for training and testing. Our organisms are *Homo sapiens* from the Animalia kingdom, *Aspergillus nidulans* from the Fungi kingdom, *Methanococcus maripaludis* from the Archaea kingdom, and *Streptococcus pneumoniae* from the Bacteria kingdom. The performance metrics—AUC, specificity, and sensitivity—are shown in Table 6.

You can see some drop off in sensitivity but overall the results are quite robust. Furthermore, given the high values of both the AUC and specificity we could easily adjust the detection threshold to bring the sensitivity into a desired range without significant degradation of the specificity.

For completeness we include the corresponding results for Glimmer in Table 7.

As might be expected we can see a considerable drop off in performance.

The MATLAB programs used to generate the results presented in this work are available on the GitHub repository, AMICodingRegionPrediction, https://github.com/gnewcombUNL/AMICodingRegionPrediction (accessed on 7 August 2021). We have also included a detailed example on how to use the program and modify parameters to accommodate the interests of the users. We have also included a MATLAB script for readers who would like to develop an annotation tool using the metrics presented here.

## 5. Conclusions

We have presented signals generated using three different measures, the average mutual information, the eaAMI, and the eAMI for differentiating between coding and noncoding regions. The AMI profile has never quite lived up to its original promise of differentiating between coding and noncoding regions since their introduction almost twenty years ago [14,16,29]. Here we show that if we remove some of the averaging effect involved in the computation of the profile the coding/noncoding signal not only becomes very strong under conditions where we use sequences from the same species for both training and testing, the signals are also highly discriminatory when the training and test sequences belong to different species. This is true even when the different species belong to different kingdoms thus in a sense redeeming the promise of twenty years. While the AMI profile has some predictive value, it is substantially outperformed by the simpler derived measure eaAMI. The signal generated by using eaAMI is strong enough to generate AUC values for a SVM classifier of greater than 0.9 in almost all cases. This is true even when the classifier is trained using DNA from an organism belonging to a different kingdom. The robustness and strength of this signal is such that it should substantially enhance the performance of current genome annotation algorithms. In particular it should be useful for annotating genomes of unknown origin. Such situations are likely to arise more often with the increasing recurrence of previously unknown infectious diseases.

## Figures and Tables

**Table 1:** Median cross-species results for all profiles using length 100 and 1000 base pair sequences.

	100 bp Sequences			1000 bp Sequences		
	**AUC**	**Sensitivity**	**Specificity**	**AUC**	**Sensitivity**	**Specificity**
eaAMI	0.788	0.606	0.837	0.991	0.904	0.985
eAMI	0.847	0.672	0.840	0.970	0.703	0.983
AMI	0.515	0.494	0.540	0.737	0.678	0.727
Glimmer	0.694	0.253	0.884	0.832	0.122	0.996

**Table 2:** Cross-species coding region prediction results for selected *Aspergillus* species using eaAMI profiles. SVMs were trained on length 1000 sequences from the species denoted in the row headers, and used to classify sequences from the species denoted in the column headers.

	*A. nidulans*			*A. fumigatus*			*A. niger*			*A. oryzae*		
**Train**	**AUC**	**SN**	**SP**	**AUC**	**SN**	**SP**	**AUC**	**SN**	**SP**	**AUC**	**SN**	**SP**
*A. nid*	0.98	0.94	0.93	0.99	0.96	0.93	0.99	0.96	0.93	0.97	0.92	0.91
*A. fum*	0.98	0.93	0.94	0.99	0.94	0.95	0.99	0.94	0.94	0.97	0.89	0.93
*A. nig*	0.98	0.95	0.92	0.99	0.96	0.92	0.99	0.96	0.93	0.97	0.92	0.90
*A. ory*	0.98	0.97	0.90	0.99	0.97	0.89	0.99	0.97	0.91	0.98	0.94	0.91

**Table 3:** Cross-species coding region prediction results for selected *Aspergillus* species using Glimmer. Glimmer was trained on length 1000 sequences from the species denoted in the row headers, and used to classify sequences from the species denoted in the column headers.

	*A. nidulans*			*A. fumigatus*			*A. niger*			*A. oryzae*		
**Train**	**AUC**	**SN**	**SP**	**AUC**	**SN**	**SP**	**AUC**	**SN**	**SP**	**AUC**	**SN**	**SP**
*A. nid*	0.93	0.91	0.76	0.91	0.92	0.71	0.89	0.84	0.76	0.90	0.88	0.83
*A. fum*	0.92	0.86	0.84	0.92	0.91	0.80	0.90	0.82	0.84	0.91	0.86	0.88
*A. nig*	0.93	0.91	0.80	0.92	0.93	0.74	0.92	0.90	0.73	0.92	0.90	0.84
*A. ory*	0.90	0.89	0.67	0.91	0.91	0.60	0.86	0.83	0.62	0.93	0.90	0.76

**Table 4:** Cross-species coding region prediction results for selected species from different orders of the Gammaproteobacteria class using eaAMI profiles.

	*A. baumannii*			*S. pneumoniae*			*S. enterica*			*V. cholerae*		
**Train**	**AUC**	**SN**	**SP**	**AUC**	**SN**	**SP**	**AUC**	**SN**	**SP**	**AUC**	**SN**	**SP**
*A. bau*	1.00	0.97	0.99	1.00	0.88	0.99	1.00	0.96	0.99	1.00	0.96	0.99
*S. pne*	1.00	0.97	0.98	1.00	0.98	0.99	0.99	0.87	0.98	0.99	0.94	0.94
*S. ent*	0.99	0.88	0.99	0.99	0.86	0.99	1.00	0.97	1.00	1.00	0.94	1.00
*V. cho*	0.99	0.95	0.97	0.99	0.88	0.99	1.00	0.97	0.98	1.00	0.98	0.99

**Table 5:** Cross-species coding region prediction results for selected species from different orders of the Gammaproteobacteria class using Glimmer.

	*A. baumannii*			*S. pneumoniae*			*S. enterica*			*V. cholerae*		
**Train**	**AUC**	**SN**	**SP**	**AUC**	**SN**	**SP**	**AUC**	**SN**	**SP**	**AUC**	**SN**	**SP**
*A. bau*	0.92	0.94	0.44	0.79	0.69	0.83	0.30	0.12	0.65	0.64	0.62	0.57
*S. pne*	0.80	0.73	0.80	0.96	0.98	0.44	0.21	0.03	0.87	0.41	0.24	0.78
*S. ent*	0.91	0.32	0.99	0.85	0.07	1.00	0.98	0.99	0.43	0.94	0.66	0.99
*V. cho*	0.96	0.77	0.97	0.93	0.54	0.98	0.91	0.88	0.76	0.98	0.98	0.59

**Table 6:** Cross-species coding region prediction results for selected highly divergent species from different kingdoms using eaAMI profiles.

	*H. sapiens*			*A. nidulans*			*M. maripaludis*			*S. pneumoniae*		
**Train**	**AUC**	**SN**	**SP**	**AUC**	**SN**	**SP**	**AUC**	**SN**	**SP**	**AUC**	**SN**	**SP**
*H. sap*	0.99	0.94	0.97	0.93	0.69	0.94	0.99	0.74	1.00	0.99	0.83	1.00
*A. nid*	0.93	0.66	0.97	0.98	0.94	0.93	1.00	0.98	0.99	0.99	0.92	0.96
*M. mar*	0.89	0.59	0.98	0.93	0.66	0.94	1.00	1.00	1.00	0.99	0.84	1.00
*S. pne*	0.94	0.86	0.89	0.92	0.93	0.70	1.00	0.98	0.99	1.00	0.98	0.99

**Table 7:** Cross-species coding region prediction results for selected highly divergent species from different kingdoms using Glimmer.

	*H. sapiens*			*A. nidulans*			*M. maripaludis*			*S. pneumoniae*		
**Train**	**AUC**	**SN**	**SP**	**AUC**	**SN**	**SP**	**AUC**	**SN**	**SP**	**AUC**	**SN**	**SP**
*H. sap*	0.71	0.95	0.04	0.50	0.08	0.93	0.88	0.36	0.93	0.77	0.34	0.99
*A. nid*	0.80	0.77	0.74	0.93	0.91	0.76	0.89	0.16	0.91	0.87	0.30	0.96
*M. mar*	0.39	0.19	0.77	0.23	0.00	0.99	0.98	0.99	0.55	0.53	0.26	0.89
*S. pne*	0.42	0.41	0.43	0.22	0.01	0.92	0.92	0.76	0.95	0.96	0.98	0.44

## Data Availability

The data and the programs used to generate the results presented in this work are available on the GitHub repository, AMICodingRegionPrediction, https://github.com/gnewcombUNL/AMICodingRegionPrediction (accessed on 7 August 2021).

## Supplementary Materials

Spreadsheets containing all the cross species prediction results for the proposed metric and Glimmer and the list of organism with taxa id and accession numbers are available online at https://www.mdpi.com/article/10.3390/e23101324/s1.
